# Vitamins and nutraceuticals in glaucoma research

**DOI:** 10.1177/11206721261419640

**Published:** 2026-02-04

**Authors:** Flora Hui, Pete A Williams

**Affiliations:** 1 Centre for Eye Research Australia, 117585 Royal Victorian Eye and Ear Hospital, Melbourne, Australia; 2 Department of Clinical Neuroscience, Division of Eye and Vision, St. Erik Eye Hospital, 27106 Karolinska Institutet, Stockholm, Sweden

**Keywords:** GLAUCOMA, glaucoma medical therapies < GLAUCOMA, optic neuropathy < NEURO OPHTHALMOLOGY, diagnostic techniques < GLAUCOMA, epidemiology / risk factors < GLAUCOMA

## Abstract

Glaucoma is a leading cause of irreversible blindness worldwide. There is no current cure for glaucoma, and it is managed by lowering intraocular pressure, a major modifiable risk factor. Nutraceuticals have long been studied to protect retinal ganglion cells and the optic nerve from degeneration by targeting the metabolic and neurodegenerative aspects of glaucoma, and even contributing to pressure lowering. A range of vitamins, minerals and nutraceuticals have been and are undergoing investigation for glaucoma. Compounds such as NAD-boosting supplements, antioxidants, and combination therapies can potentially support retinal ganglion cell metabolism and reduce oxidative stress, leading to healthier neurons to preserve vision for longer. However, varying levels of evidence exist to support the use of nutraceuticals in glaucoma. Herein lies a comprehensive review on the latest research evidence for 35 different vitamins, minerals, and nutraceuticals, their proposed mechanism of action, and reported effects on retinal ganglion cell health and in people with glaucoma.

## Introduction

Glaucoma is a common neurodegenerative disease characterized by the degeneration of retinal ganglion cells and their axons, leading to characteristic structural changes at the optic nerve head and corresponding visual field loss. Despite being a multifactorial disease with a complex etiology, the unifying feature of glaucoma is retinal ganglion cell degeneration, which often results in irreversible blindness if left untreated. Importantly, while elevated intraocular pressure (IOP) is a well-established risk factor and the only available treatment target, it is neither necessary nor sufficient for the development of glaucoma, as evidenced by the existence of normal-tension glaucoma and the observation that many patients continue to progress despite achieving target IOP levels.^1,2^

The prevalence of glaucoma increases with age, making it a significant global health burden as populations age. It is estimated that the number of individuals affected by glaucoma will continue to rise, with substantial socioeconomic impact due to vision loss and associated disability.^1,3^ Age is the most prominent risk factor, but genetic predisposition, vascular dysregulation, and systemic metabolic dysfunction have also been implicated in disease susceptibility and progression. Recent genomic and metabolomic studies have revealed that mitochondrial dysfunction and impaired energy metabolism are central to the vulnerability of RGCs in glaucoma, highlighting the importance of bioenergetic support as a therapeutic target.^4–6^

Current clinical management of glaucoma is centered on lowering IOP through topical medications, laser therapy, or surgical interventions. While these approaches are effective in slowing disease progression in many patients, up to 40% still develop significant vision loss or blindness in at least one eye over their lifetime, underscoring the need for additional therapeutic strategies.^2^ Notably, IOP-lowering therapies do not directly address the underlying neurodegeneration of retinal ganglion cells, and there are currently no clinically approved neuroprotective treatments that target the retina or optic nerve directly.

Diagnosis of glaucoma relies on a combination of structural and functional assessments, including optical coherence tomography (OCT) to measure retinal nerve fiber layer thickness, and standard automated perimetry to detect visual field defects. However, these modalities typically detect damage only after substantial RGC loss has already occurred. Studies suggest that up to 28% of retinal ganglion cells may be lost before early visual field defects become apparent.^7^ Advances in imaging, home-based visual field monitoring, and artificial intelligence-assisted analysis are being developed to improve early detection and risk stratification, but challenges remain in predicting individual disease trajectories. Although glaucoma is highly hereditary, monogenic glaucoma is rare, with polygenic risk more at play. Polygenic risk scores are being developed which may help categorize patients into high and low risk bins. Whilst promising, research on polygenic risk scores remains in the experimental stage and requires validation across ancestrally diverse populations and longitudinal cohorts to determine whether their predictive utility can effectively inform early intervention and management of high-risk individuals.

Given the limitations of current therapies and diagnostic tools, there is a growing focus on developing new treatments that target the metabolic and neurodegenerative aspects of glaucoma. Research has identified that retinal ganglion cells exist in a state of metabolic vulnerability, particularly with advancing age and/or in the presence of elevated IOP.^4,8^ Strategies aimed at supporting mitochondrial function and cellular metabolism including supplementation with nicotinamide (the amide form of vitamin B_3_) have demonstrated robust neuroprotective effects in preclinical models and early-phase clinical trials, improving retinal ganglion cell function and visual outcomes.^9,10^ Other neuroprotective candidates, including citicoline, coenzyme Q_10_, and dietary interventions like the ketogenic diet, are also under investigation, though large-scale clinical evidence remains limited.^4,5,11^ In addition to targeting cellular metabolism and reducing oxidative stress, some nutraceuticals also have an IOP lowering effect, which may provide further neuroprotective effects in glaucoma.

This comprehensive review covers 35 different vitamins, minerals, and nutraceuticals that have been investigated for glaucoma prevention or treatment. The evidence varies from robust clinical trial data to preliminary metabolomic findings, reflecting the diverse approaches being pursued in nutritional glaucoma research (these are overviewed in Tables 1 and 2). Many successful supplement formulations combine multiple compounds, suggesting synergistic effects may be more important than individual nutrient supplementation.

**Table 1.** Overview of evidence for vitamin and nutraceutical use in glaucoma.

Vitamin/compound	Type of studies	Key findings	Side effect profile
Vitamin A (Retinol)	Dietary/epidemiological, genetic studies	Protective association with glaucoma; lower plasma levels in patients	Nausea, headache, hair loss, cracked skin (chronic high doses)
Vitamin D (Calciferol)	Clinical, preclinical, epidemiological	Mixed results; some protective effects, optimal threshold ∼50 nmol/L	Nausea, constipation, kidney stones (chronic high doses)
Vitamin E (Tocopherol)	Dietary studies, preclinical	Lower plasma levels in glaucoma patients; potential IOP-lowering effects	Increased bleeding risk (>1,000 mg/day)
Vitamin K_1_ (Phylloquinone)	Preclinical	Protects retinal ganglion cells, reduces inflammation and ferroptosis	Generally non-toxic, rare allergic reactions
Vitamin B_1_ (Thiamine)	Dietary/epidemiological	Protective association in dietary studies; part of B-complex supplements	Strong safety profile, rare allergic reactions with IV
Vitamin B_2_ (Riboflavin)	Dietary/epidemiological	Protective association in males; nonlinear relationship in females	Strong safety profile
Vitamin B_3_ (as Nicotinamide)	Clinical trials, preclinical	Multiple ongoing randomized controlled trials; neuroprotective effects, improves retinal function	Strong safety profile, single case of hepatotoxicity in controlled clinical trial setting when combined with pyruvate
Vitamin B_5_ (Pantothenic acid)	Limited preclinical	Included in some B-complex formulations	Generally safe
Vitamin B_6_ (Pyridoxine)	Dietary studies, clinical trials	Protective association; part of current clinical trials	Dose-dependent peripheral neuropathy
Vitamin B_7_ (Biotin)	Very limited	Mentioned in supplement formulations only	Generally safe
Vitamin B_9_ (Folate)	Clinical case reports, dietary studies	Potential for normal tension glaucoma; part of B-vitamin combinations	Nausea, bloating
Vitamin B_12_ (Cobalamin)	Dietary studies, case reports	Protective trends; deficiency linked to optic neuropathy	Strong safety profile, rare allergic reactions
Vitamin C (Ascorbic acid)	Genetic studies, dietary studies	Lower plasma levels in glaucoma; genetic polymorphisms affect risk	Kidney stones (chronic high doses), GI upset
Selenium	Cross-sectional, case-control studies	Mixed findings; lower serum levels in patients but complex risk relationships	Hair loss, nausea, ‘garlic breath’ (chronic high doses)
Zinc	Dietary studies, serum analyses	Protective association; lower serum concentrations in glaucoma patients	Nausea, copper deficiency (chronic high doses)
Copper	Limited dietary studies	Protective effects through oxidative stress modulation	Hepatotoxicity, GI upset
Magnesium	Population studies, clinical formulations	Conflicting findings; included in successful neuroprotective formulations	Diarrhea, hypotension
Lutein/zeaxanthin	Clinical trials, systematic reviews	Neuroprotective benefits; increases serum carotenoid concentrations	Strong safety profile
Beta-carotene	AREDS-related studies	Investigated as part of AREDS formula for glaucoma applications	Harmless skin yellowing, increased lung cancer risk (in smokers)
Taurine	Preclinical studies	Neuroprotective properties for retinal ganglion cells	Strong safety profile, rare nausea or headache
Homotaurine	Clinical trials	Multiple successful trials in combination formulations; slows visual field progression	GI upset
Carnosine	Clinical formulations	Antioxidant properties; component of successful supplement formulations	Typically well-tolerated
Resveratrol	Meta-analyses, clinical trials	Enhances retinal ganglion cell survival; multiple neuroprotective mechanisms	GI upset
Curcumin	Preclinical, limited clinical	Anti-inflammatory properties; potential for glaucoma progression reduction	GI upset, drug interactions
Anthocyanins/bilberry	Clinical studies	Improved eye blood flow, decreased IOP, reduced visual field loss	Typically well-tolerated
Nicotinamide riboside	Preclinical, clinical trials	Increases retinal NAD levels; preserves retinal ganglion cell function	Typically well-tolerated
Alpha-Lipoic acid	Preclinical studies	Enhanced antioxidant gene expression; retinal ganglion cell protection	Headache, GI upset, skin rash
Melatonin	Clinical trials, preclinical	IOP reduction and neuroprotection	Drowsiness, headache, drug interactions
Forskolin	Clinical formulations, preclinical	IOP-lowering and neuroprotective; component of successful combinations	Hypotension, tachycardia
EGCG/whole green tea polyphenols	Preclinical	Promotes NAD production; mentioned in neuroprotection studies	Hepatotoxicity (chronic high doses)
Ginkgo biloba	Clinical trials, systematic reviews	Mixed results; insufficient evidence for efficacy	Headache, GI upset
Coenzyme Q_10_	Preclinical, clinical studies	Reduces glial activation; neuroprotective when combined with citicoline	GI upset, insomnia (high doses later in the day)

**Table 2.** Overview of ongoing clinical studies for vitamins and nutraceuticals in glaucoma.

Trial Title	Clinical Trial ID	Phase	Status	Condition	Intervention	Location	Participants
*Nicotinamide Trials*							
Targeting Metabolic Insufficiency (TAMING) Glaucoma Study	NCT06731582	Phase III	Recruiting	Glaucoma	Nicotinamide 1.5 g daily	Singapore (National University Hospital, Singapore Eye Research Institute)	520
The Glaucoma Nicotinamide Trial (TGNT)	NCT05275738	Phase III	Recruiting	Glaucoma	Nicotinamide 1.5–3.0 g daily	Sweden (Umeå University, St. Erik Eye Hospital Stockholm)	660
Vitamin B_3_ in Glaucoma Study (VBIGS)	ANZCTR: ACTRN12622000414718	Phase III	Recruiting	Glaucoma	Nicotinamide 1.5–3.0 g daily	Australia (Centre for Eye Research Australia, University of Adelaide)	424
Nicotinamide in Glaucoma (NAMinG): A Randomized, Placebo-controlled, Multi-center, Phase III Trial	NCT05405868	Phase III	Recruiting	Glaucoma	Nicotinamide 1.5–3.0 g daily	United Kingdom	496
Nicotinamide and Pyruvate for Open Angle Glaucoma	NCT05695027	Phase II	Recruiting	Open-angle glaucoma	Nicotinamide + Pyruvate	United States	42
*NAD Precursor Trials*							
Comparisons of NAD Precursors for Neuroenhancement in Glaucoma	NCT06991712	Phase II	Recruiting	Glaucoma	Nicotinamide Riboside, Nicotinamide, NMN, Nicotinic Acid	Hong Kong (HKU Eye Centre)	115
Nicotinamide Riboside as Neuroprotective Therapy for Glaucoma	Chinese Clinical Trial Registry 1900021998	Phase II	Recruiting	Primary open-angle glaucoma	Nicotinamide riboside 300mg	Multiple centers	125
*B-Vitamin Combination Trials*							
Vitamin Mix (B6, B9, B12, and Choline) for Glaucoma Patients (IVMGS)	NCT06885827	Phase II	Recruiting	Open-angle glaucoma, Pseudoexfoliation glaucoma	B6, B9, B12, Choline	Sweden (Stockholm - St Eriks Ögonsjukhus, Karolinska Institutet)	80
*Biomarker and Mechanistic Studies*							
Nicotinamide Levels in Serum, Aqueous Humor, and Tear Film in Glaucoma	NCT07006194	N/A	Not yet recruiting	Glaucoma	Nicotinamide tablet	Italy (Chieti, Pisa, Sassari)	360
Lutein, Zeaxanthin, and Omega-3 Clinical Trial	Thai Clinical Trials Registry 20220904002	Phase III/IV	Recruiting	Digital eye strain	Lutein, zeaxanthin, omega-3	Multiple centers	600

## Fat-soluble vitamins

### Vitamin A (retinol)

Vitamin A represents one of the most critical nutrients for eye health, functioning as both a structural component of visual pigments and a protective antioxidant. Vitamin A exists in two primary forms: retinol from animal sources and provitamin A carotenoids from plant sources, with beta-carotene being the most prevalent and effective provitamin A carotenoid.^12^ Vitamin A plays a fundamental role in maintaining corneal health, helping to protect the surface of the eye. Dietary studies consistently show protective associations with glaucoma, with a systematic review and meta-analysis demonstrating that dietary vitamin A intake had an odds ratio of 0.45 for open-angle glaucoma after addressing heterogeneity.^13,14^

### Vitamin D (calciferol)

Vitamin D has emerged as an important nutrient for eye health, with research suggesting it may help prevent or delay various ocular conditions through its antioxidant and anti-inflammatory properties. Its protective mechanisms appear to involve offsetting oxidative stress that contributes to the development of chronic eye diseases, including age-related macular degeneration (AMD), cataracts, and diabetic retinopathy.^15,16^ A large-scale UK Biobank study of over 322,000 participants identified 50 nmol/L as the optimal serum 25(OH)D threshold for reducing risks of cataract, AMD, and diabetic retinopathy, though no significant association with primary open-angle glaucoma was observed.^17^ However, preclinical studies demonstrate that vitamin D3 supplementation can reduce inflammatory markers in the trabecular meshwork and provide neuroprotective effects. The levels of vitamin D in aqueous humor were notably lower in patients with both cataracts and glaucoma compared to those with cataracts alone. Mendelian randomization analyses found no statistical evidence that vitamin D levels or deficiency causally affect the risk of developing primary open-angle glaucoma.^18^ These results indicate that vitamin D may not be a significant factor in modifying primary open-angle glaucoma risk and challenges the above data that vitamin D supplementation could be effective in reducing the risk of glaucoma.

### Vitamin E (tocopherol)

Vitamin E shows potential in glaucoma management through its antioxidant and neuroprotective properties.^19^ Studies have demonstrated that glaucoma patients have statistically significant lower plasma vitamin E concentrations compared to controls.^20^ Recent investigations into mixed tocopherol and tocotrienol combinations show effectiveness in reducing IOP in animal models within 2 days of treatment. The neuroprotective and antioxidative properties of vitamin E derivatives make them promising candidates for glaucoma management. Clinical trials have evaluated vitamin E in combination with other compounds for glaucoma treatment. A fixed combination of citicoline 500 mg, homotaurine 50 mg, and vitamin E 12 mg demonstrated beneficial effects on contrast sensitivity and visual-related quality of life in patients with primary open-angle glaucoma.^21^

### Vitamin K (phylloquinone K_1_)

Vitamin K_1_ plays a protective role in ocular health through its antioxidant and anti-inflammatory properties, including the regulation of microglial ferroptosis and the upregulation of matrix Gla protein, which is essential for IOP regulation and trabecular meshwork integrity. Higher dietary intake of vitamin K_1_ has been associated with a reduced risk of cataract surgery and may offer broader neuroprotective benefits within the visual system.^22^ Vitamin K_1_ represents an emerging area of glaucoma research with particularly promising preclinical results. Recent studies demonstrate that vitamin K_1_ can alleviate retinal inflammation following acute ocular hypertension by modulating microglial iron overload and ferroptosis.^23^ High-dose vitamin K_1_ intake inhibits retinal ganglion cell loss during glaucomatous injury, potentially through two mechanisms: lowering IOP and directly counteracting glaucoma-induced cellular damage through neuroprotective effects. This vitamin shows capability to regulate ferroptosis pathways, which are increasingly recognized as important in glaucoma pathogenesis.^22^

## Water-soluble B-complex vitamins

### Vitamin B_1_ (thiamine)

Vitamin B_1_ plays a crucial role in ocular health by supporting corneal nerve integrity and function, with supplementation shown to improve corneal nerve parameters and alleviate symptoms in dry eye disease.^24^ Higher dietary intake of vitamin B_1_ is associated with a reduced risk of AMD and may also help protect against cataracts and uveitis.^25^ Thiamine has shown protective associations in dietary epidemiological studies of glaucoma.^13,26,27^ Metabolomic profiling of aqueous humor from glaucoma patients revealed significantly decreased thiamine levels compared to controls.^28^ Experimental studies demonstrate that thiamine treatment (alone or as part of a vitamin combo) significantly reduced inflammatory response and protected retinal ganglion cell function against neuroinflammatory damage in mouse models.^28,29^ Both agmatine and thiamine treatment significantly attenuated Toll-like receptor agonist-induced proinflammatory cytokine responses and rescued pattern electroretinogram amplitudes in treated groups.

### Vitamin B_2_ (riboflavin)

Riboflavin plays a critical role in eye health, with deficiency leading to structural and functional damage to the retina and ocular surface, including retinal degeneration and corneal abnormalities. Supplementation with riboflavin has shown benefit in eye disease, such as improving retinal function in riboflavin transporter deficiency and is being used in corneal cross-linking procedures for conditions like keratoconus.^30–32^ Riboflavin is included in various B-complex supplement formulations that have demonstrated efficacy in preclinical glaucoma models.^33^ Its role in cellular energy metabolism and antioxidant function provides biological plausibility for its protective effects.

### Vitamin B_3_ (niacin, nicotinamide, nicotinamide riboside)

Nicotinamide adenine dinucleotide (NAD) is an essential metabolite and neuroprotective molecule, often referred to as the “longevity molecule”. NAD supports cellular metabolism by acting as a cofactor for hundreds of enzymatic reactions and serving as a key regulator of neurodegeneration.^34^ NAD is produced in the body through three primary routes: *de novo* from tryptophan, from niacin in the Preiss-Handler pathway, and from nicotinamide in the salvage pathway (with an alternate entry by nicotinamide riboside). Niacin (nicotinic acid), nicotinamide (niacinamide), and nicotinamide riboside are all different vitamers of vitamin B_3_ but should not be confused since their routes to generate NAD, neuroprotective properties, and side effect profiles all differ.

The capacity to maintain NAD levels in the retina and optic nerve declines with age, which increases the vulnerability of retinal ganglion cells to pressure-related stress, leading to dysfunction and degeneration.^10,35^ Preventing age-related NAD depletion, via administration of nicotinamide^10,36^ (the amide form of vitamin B_3_ and a precursor to NAD) or via gene therapy^10,37^ (targeting enzymes involved in NAD production), has demonstrated robust protection against metabolic decline and neurodegeneration in animal models of glaucoma. At the highest doses tested, nicotinamide also lowered IOP by 1 mmHg in control (but not ocular hypertensive) eyes. In patients with glaucoma, systemic nicotinamide is low,^38^ NAD levels are significantly reduced in peripheral blood mononuclear cells and in the serum,^39^ and key enzymes for producing NAD are low in post mortem retinas.^40^ Population studies have also pointed to low niacin in glaucoma populations.^41^ Supplementation with nicotinamide improves visual function in existing glaucoma patients^9^ supporting the hypothesis that glaucoma has a strong metabolic component and that correcting these bioenergetic insufficiencies can provide neuroprotection in animal models and improve vision in at least some patients with glaucoma.

Nicotinamide riboside is another precursor to NAD with early evidence of protection in glaucoma. Rodent models of retinal ganglion cell injury demonstrated that high dose oral supplementation of nicotinamide riboside could prevent axonal degeneration,^42^ and enhance cell survival. Preservation of retinal function was also observed in the acute optic nerve crush model.^43^ There is currently one randomized controlled trial investigating the utility of low-dose nicotinamide riboside in glaucoma to prevent retinal nerve fiber layer loss.^44^

### Vitamin B_6_ (pyridoxine)

Metabolomic studies have identified vitamin B_6_ among compounds altered in glaucoma patients’ aqueous humor.^45^ Vitamin B_6_ deficiency can cause optic neuropathy that is reversible if identified and treated promptly, emphasizing its importance for optic nerve health.^46^ Current clinical trials are investigating combinations of vitamins B_6_, B_9_, and B_12_, and choline for neuroprotective effects in glaucoma.

### Vitamin B_9_ (folate)

Folate is essential for DNA synthesis, cell division, and the formation of healthy red blood cells. Adequate folate levels are important for the health of the retina and optic nerve, and deficiency can lead to optic neuropathy and increase the risk of retinal vascular diseases and AMD.^47–49^ Research supports that folate supplementation may help protect against certain eye diseases, particularly those associated with elevated homocysteine levels or vascular dysfunction in the retina. Folate research in glaucoma is emerging as particularly relevant for normal tension glaucoma. Recent clinical approaches propose using L-methylfolate and methylcobalamin supplementation to address elevated retinal venous pressure, which impedes retinal perfusion and contributes to oxidative stress in normal tension glaucoma.^50^ Folate was identified through an RNA-sequencing screen of retinal ganglion cell injury models and demonstrated to provide neuroprotection in a retina axotomy model.^51^ Current clinical trials are investigating the combination of vitamins B_6_, B_9_, and B_12_ with choline, showing promising results in animal models.^45^

### Vitamin B_12_ (cobalamin)

Vitamin B_12_ is essential for DNA synthesis, red blood cell formation, and the maintenance of neuronal function.^52^ It plays a critical role in eye health by supporting the optic nerve and protecting against neurodegeneration.^53,54^ Deficiency leads to optic neuropathy, which may cause progressive and potentially irreversible vision loss if not promptly treated.^54^ Adequate vitamin B_12_ levels may help reduce the risk of AMD and other ocular diseases by lowering homocysteine and supporting retina and optic nerve health.^55^ Vitamin B_12_ deficiency has been linked to optic neuropathy, with cases demonstrating that untreated deficiency can lead to irreversible optic nerve damage.^56^ Vitamin B_12_ is included in current clinical trials investigating B-vitamin combinations for glaucoma neuroprotection.^45^

### Vitamin C (ascorbic acid)

Vitamin C has been studied extensively in glaucoma through both genetic and dietary approaches. A Mediterranean population study found that glaucoma patients had significantly lower plasma vitamin C concentrations compared to controls, and genetic polymorphisms in vitamin C transporter genes were associated with glaucoma risk.^20,57,58^ The corneal epithelium and lens have particularly high levels of vitamin C, and deficiency can impair corneal wound healing.^59,60^ Recent dietary studies show that higher quartiles of vitamin C intake were associated with lower odds of cataracts and glaucoma compared to the lowest quartile.^61–63^ Vitamin C has also been demonstrated to be neuroprotective and anti-neuroinflammatory in mouse models of ocular hypertension, with one report suggesting that these effects are exerted through SPP1.^64,65^

## Minerals and trace elements

### Copper

Copper has an essential role in retinal physiology, supporting antioxidant enzyme function and retinal cell survival. Copper deficiency is linked to optic neuropathy and AMD, with studies showing reduced copper levels in the retinal pigment epithelium and choroid complex of AMD patients. Supplementation with copper, often alongside zinc, has been associated with a reduced risk of AMD progression.^66–69^ In glaucoma, several studies have found that copper levels are elevated in the aqueous humor of glaucoma patients compared to controls, and this increase is often accompanied by a lower zinc level.^70^ Other groups have observed a negative correlation between serum zinc and copper levels in glaucoma patients, suggesting that an increased copper-to-zinc ratio may be relevant in glaucoma pathophysiology.^71^ Further research is required to determine whether copper is a causative factor or a biomarker of disease progression.

### Magnesium

Magnesium is an essential mineral present in high concentrations in ocular tissues such as the cornea, lens, and retina, where it supports cellular metabolism, antioxidant defense, and ionic balance. Adequate magnesium levels are crucial for maintaining the structural and functional integrity of the eye, with deficiency linked to cataract, diabetic retinopathy, and glaucoma. Magnesium also exhibits neuroprotective effects by reducing oxidative stress and regulating calcium influx, which helps protect retinal ganglion cells.^72^ In primary open-angle glaucoma, magnesium levels in ocular tissues and serum are significantly lower compared to healthy controls.^73^ Magnesium supplementation may improve visual fields, enhance ocular blood flow, and reduce IOP in glaucoma patients, likely due to its vasodilatory, antioxidant, and neuroprotective properties.^72,73^ Supporting a role for magnesium in glaucoma, in a small clinical trial, oral magnesium therapy improved visual field parameters in glaucoma patients with vasospasm.^74^

### Selenium

Selenium is an essential trace mineral that functions as a key component of antioxidant enzymes (selenoproteins) which protect from oxidative damage. In the eye, selenium's antioxidant and anti-inflammatory properties may help prevent or delay cataract, AMD, and thyroid eye disease by counteracting oxidative stress and supporting ocular tissue health. Clinical studies suggest that selenium supplementation can reduce the severity and progression of mild thyroid eye disease, especially in populations with low selenium status.^75,76^ Current evidence suggests a potential association between higher selenium levels and an increased risk of glaucoma, though causality has not been established. A large cross-sectional study using NHANES data from US adults aged 40 and older found that higher dietary selenium intake was associated with an increased risk of glaucoma (odds ratio 1.39; 95% CI, 1.07–1.81), with a linear relationship observed across subgroups.^77^ A case-control study measuring selenium in plasma and aqueous humor of patients with primary open-angle glaucoma and controls reported that individuals in the highest tertile of plasma selenium had a significantly higher odds of glaucoma (OR = 11.3), while those in the middle tertile of aqueous humor selenium had a significantly lower odds (OR = 0.06).^78^

### Zinc

Zinc is highly concentrated in the retina, where it plays a critical role in supporting retinal health and enabling vitamin A to produce melanin. Adequate zinc intake is associated with a reduced risk of AMD and may help slow its progression, while zinc deficiency can impair night vision and contribute to optic neuropathy.^63,66,67,79,80^ In glaucoma, the Composite Dietary Antioxidant Index study demonstrated that higher quartiles of zinc intake were associated with significantly lower odds of glaucoma (OR: 0.73).^63^ Serum zinc concentrations are notably lower in glaucoma patients compared to healthy controls.^81^

## Other commonly explored nutritional supplements / nutraceuticals

### Alpha-lipoic acid

Alpha lipoic acid is a potent antioxidant that protects ocular tissues by neutralizing free radicals and regenerating other antioxidants, contributing to the maintenance of retinal and vascular health in the eye. There is evidence for alpha-lipoic acid in diabetic retinopathy and dry eye disease by reducing oxidative stress and inflammation, and supporting nerve function and blood flow in ocular tissues.^82^

Supporting this in glaucoma, in mouse animal models of glaucoma, dietary alpha-lipoic acid reduces oxidative stress, upregulates antioxidant enzymes, and provides retinal ganglion cell neuroprotection.^83^ Novel liposomal formulations have been developed to improve alpha-lipoic acid bioavailability for glaucoma treatment.^84^

### Coenzyme Q_10_

Coenzyme Q_10_ is a vital mitochondrial antioxidant present in high concentrations in the retina, where it protects from oxidative stress and supports cellular energy production. Coenzyme Q_10_ levels decline with age, and this may contribute to AMD and optic neuropathies. Supplementation with coenzyme Q_10_ has demonstrated promise in experimental models for reducing retinal cell apoptosis and supporting overall retinal health.^85,86^ Coenzyme Q_10_ has demonstrated neuroprotective effects in both animal models and human studies of glaucoma, primarily by reducing oxidative stress and inhibiting retinal ganglion cell apoptosis. Both topical or dietary coenzyme Q_10_ can promote retinal ganglion cell survival, preserve optic nerve axons, and reduce markers of oxidative stress and apoptosis in glaucomatous eyes.^86–92^ Early clinical studies indicate that coenzyme Q_10_, especially when combined with vitamin E, may improve retinal function and visual cortical responses in glaucoma patients.^93–95^

### Curcumin

Curcumin is the primary active compound derived from the rhizome of *Curcuma longa* (turmeric) and is a yellow polyphenolic compound that exhibits potent antioxidant, anti-inflammatory, and neuroprotective properties. Curcumin has demonstrated significant therapeutic potential through multiple mechanisms including inhibition of oxidative stress, modulation of inflammatory pathways, and protection against vascular endothelial growth factor-mediated angiogenesis. Research has demonstrated effectiveness in AMD, diabetic retinopathy, dry eye disease, and retinal degeneration. Several promising preclinical studies have investigated the potential neuroprotective effects of curcumin in glaucoma. A significant breakthrough was the development of a novel nanocarrier formulation that dramatically improves curcumin's bioavailability.^96^ Topical curcumin nanocarrier eye drops demonstrated significant neuroprotective effects by reducing retinal ganglion cell loss compared to controls (ocular hypertension and optic nerve transection). Additional preclinical research has demonstrated that curcumin protects retinal ganglion cells through multiple mechanisms including restoring NF-κB expression,^97^ preventing oxidative damage, and maintaining mitochondrial function whilst regulating antioxidant systems in ischemia-reperfusion injury models.^98,99^ Curcumin protects trabecular meshwork cells from oxidative stress-induced damage,^100^ reducing inflammatory markers, and cellular apoptosis. Whilst preclinical findings are encouraging, clinical trials remain limited (although there are registered studies investigating curcumin's effects on intraocular pressure and ocular surface parameters in glaucoma patients).

### Epigallocatechin gallate (EGCG)

EGCG is a major component of the green tea polyphenols. It is a powerful antioxidant with anti-inflammatory, neuroprotective, and anti-angiogenic properties, making it a promising therapeutic candidate.^101^ Preclinical studies have demonstrated strong neuroprotective effects of EGCG in glaucoma models^37,101,102^ via oral or intraperitoneal routes. A recent study demonstrated EGCG as neuroprotective in ocular hypertensive rats, and was able to isolate a primary effect NAD production (although other mechanisms may remain and be viable to provide neuroprotection including via NF-κB).^37,103,104^ Subsequent work developed EGCG analogues also demonstrated strong neuroprotective potential.^37^ Clinical evidence is limited but promising, with a randomized placebo-controlled trial showing that EGCG supplementation (200 mg/day) for 3 months improved pattern-evoked electroretinogram amplitudes in patients with primary open-angle glaucoma, particularly those with early to moderately advanced disease.^105^ Additionally, a recent study demonstrated that both green tea extract and EGCG (400 mg capsules) significantly reduced intraocular pressure in healthy volunteers.^106^ EGCG has demonstrated strong neuroprotection in animal models, however its poor viability may explain its lack of success in clinical trials.

### Forskolin

Forskolin is a natural diterpene compound derived from the roots of *Coleus forskohlii* (a plant in the mint family native throughout India and Southeast Asia). Forskolin functions as a potent activator of adenylyl cyclase increasing intracellular cAMP levels Forskolin has demonstrated significant therapeutic potential primarily through its ability to reduce IOP by decreasing aqueous humor production, making it a promising candidate for further glaucoma research. Current research supports forskolin exhibiting neuroprotective properties and anti-inflammatory effects. *In vitro* forskolin induced a two-fold increase in retinal ganglion cell survival in mixed retinal cell cultures after 48 h, with this neuroprotective effect mediated through cAMP-dependent mechanisms involving protein kinase A activation.^107^ In retinal ischemia-reperfusion injury models, forskolin eye drops (10 μM) significantly reduced neuronal and vascular damage whilst decreasing inflammatory markers.^108^ Intravitreal injections of a nutraceutical mix containing forskolin, homotaurine, and L-carnosine demonstrated synergistic neuroprotective effects of the three-compound combination through PI3 K/Akt pathway upregulation.^109^ Further supporting this, a nutraceutical mix study by Locri et al. demonstrated that a supplement containing forskolin, homotaurine, spearmint extract, and B vitamins prevented the reduction of photopic negative response amplitude and provided neuroprotection in a mouse optic nerve crush model of glaucoma.^110^ Clinical evidence supports these preclinical findings, with an open-label study of 90 patients demonstrating that 1% forskolin eye drops reduces IOP by 4–5 mmHg compared to baseline.^111,112^ Oral forskolin combined with rutin (a flavonoid found in citrus fruit) has shown promise in clinical trials, with patients under maximum tolerated medical therapy experiencing a further 10% reduction in IOP when supplemented with forskolin-containing formulations.^113^

### Ginkgo biloba

*Ginkgo biloba* is a tree species native to China with medicinal use for over 2,000 years. The standardized extract (EGb 761) contains ∼24% flavonoid glycosides, 6% terpene lactones (including ginkgolides), and other bioactive compounds that, together, exhibit potent antioxidant, anti-inflammatory, and neuroprotective properties. Multiple studies have shown significant dose-dependent increases in retinal ganglion cell survival following optic nerve injury. EGb 761 treatment reduces retinal ganglion cell loss in a rat model of chronic moderately elevated intraocular pressure.^114^ Subsequent dose-response studies demonstrated that retinal ganglion cell survival rates increased significantly with low, medium, and high doses of *Ginkgo biloba* extract.^115^ Clinical evidence for *Ginkgo biloba* is more variable but with some promising findings. Quaranta et al. reported significant improvements in visual field indices in normal tension glaucoma patients treated with 120 mg/day of ginkgo biloba extract for 4 weeks.^116^ A long-term Korean study demonstrated that 160 mg/day of ginkgo biloba extract significantly slowed the progression of visual field damage in normal tension glaucoma patients over an average follow-up period of 12.3 years.^117^ However, subsequent studies have shown mixed results, with some failing to replicate these beneficial effects on visual field parameters.^118^

### Glucose, lactate, and pyruvate

Retinal energy demands are met by glycolysis (2 ATP per glucose) and oxidative phosphorylation (≈32 ATP), with pyruvate and lactate serving as critical substrates. *In vitro*, retinal ganglion cells can utilize glucose, lactate, or pyruvate interchangeably.^8^ Harder et al. demonstrated a glycolytic block in the DBA/2J mouse model of ocular hypertensive glaucoma. Supporting this, oral pyruvate supplementation preserved metabolic homeostasis, reduced optic nerve degeneration, maintained axonal transport, and improved visual function.^119^ These protective effects extended to a rat laser-induced ocular hypertension model and *ex vivo* axotomy explants. Combining low-dose nicotinamide (*see above*) with pyruvate provided synergistic protection, enhancing retinal ganglion cell axon survival beyond either treatment alone.^119^ Early clinical translation includes 50% glucose eye drops that improved contrast sensitivity in open-angle glaucoma patients^120^ and a Phase II trial of combined pyruvate (3 g/day) and nicotinamide (3 g/day) demonstrating improved visual field metrics without adverse effects.^121^ Clinically, oral pyruvate is well tolerated (aside from diarrhea at >30 g/day). Early human trials using 50% glucose eye drops in open-angle glaucoma improved contrast sensitivity, suggesting that boosting retinal energy substrates could support both neuroprotection and recovery in glaucoma patients. Lactate, like pyruvate, fuels retinal ganglion cells via monocarboxylate transporters (MCT1–2) across the blood–brain and blood–retinal barriers. High levels of mitochondrial pyruvate carriers (MPC1/2) further support their reliance on both metabolites, which protect cells even under glucose deprivation. In animal glaucoma models, decreased retinal lactate and MCT2 levels coincide with injury, while MCT2 overexpression or pyruvate treatment restores neuroprotection; blocking MCTs negates pyruvate's benefits, highlighting glycolysis as a therapeutic target.^119,122^ Future challenges include safe delivery of metabolic substrates, cell-specific metabolic profiling, and leveraging gene- and CRISPR-based approaches to optimize glycolysis in human glaucoma.^123^

### Lutein and zeaxanthin

These macular carotenoids have substantial research in glaucoma, despite being more commonly associated with AMD. Following 18-month supplementation of open-angle glaucoma patients with macular pigment carotenoids (lutein, zeaxanthin and meso-zeaxanthin) in the European Nutrition in Glaucoma Management trial, Raman spectroscopic analysis confirmed increased serum carotenoid concentrations.^124,125^ Macular pigment optical density's implications in diabetic retinopathy and glaucoma, have been explored, underscoring the need for clinical measurement.^126^ A randomized controlled trial demonstrated that pistachio consumption (a rich source of xanthophyll lutein; 57 g/day for 12 weeks) significantly increased macular pigment optical density in healthy adults selected for habitually low lutein and zeaxanthin intake.^127^

### Melatonin

Melatonin has substantial research in glaucoma covering both intraocular pressure regulation and neuroprotection.^128^ Recent advances include the development of near-infrared activated liposomes for controlled melatonin delivery to the retina.^129^ This remotely triggered delivery system can decrease intraocular pressure elevation by 24%, enhance retinal ganglion cell survival by 77%, and decrease glial fibrillary acidic protein activation by 75% in experimental glaucoma models. Ketorolac, melatonin and latanoprost tri-loaded PLGA microspheres have been developed for sustained drug release over 70 days, showing promise for treating glaucoma through combined hypotensive, antioxidant and anti-inflammatory activity.^130^ Melatonin demonstrates integrative mechanisms of intraocular pressure control and neuroprotection, making it a multi-target candidate for glaucoma treatment.

### Resveratrol

Resveratrol is a natural polyphenolic stilbene predominantly known for its high concentrations in grape skins. It exhibits potent antioxidant, anti-inflammatory, anti-angiogenic, and neuroprotective effects.^131–135^ Preclinical studies demonstrate that resveratrol protects retinal ganglion cells and lowers IOP through multiple mechanisms. Topical trans-resveratrol (0.2%) reduced IOP by up to 25% in steroid-induced ocular hypertension via agonism at adenosine A₁ receptors. In rodent models of acute IOP elevation and optic nerve crush, systemic or intravitreal resveratrol treatment (10–250 mg/kg or 30 µM) provided neuroprotection, preserved retinal thickness, upregulated SIRT1 expression, and inhibited pro-inflammatory and apoptotic markers. A systematic meta-analysis of 30 preclinical glaucoma studies found that resveratrol increased retinal ganglion cell survival and slowed retinal thinning.^136–138^ Although human clinical trials are limited, these data provide a robust foundation for advancing resveratrol research in glaucoma.

### Taurine and homotaurine

Taurine is a conditionally essential amino acid highly concentrated in the retina, where it plays a crucial role in maintaining retinal structure, protecting photoreceptors, and supporting antioxidant defenses. Deficiency in taurine leads to retinal degeneration, oxidative stress, and loss of retinal ganglion cells, while supplementation has been shown to protect against light-induced and age-related retinal damage. Taurine's neuroprotective, anti-inflammatory, and anti-apoptotic properties make it important for ocular health.^139–141^ Metabolomic studies demonstrate reduced taurine concentrations in the eyes of glaucoma patients, supporting the hypothesis that taurine depletion may contribute to disease progression.^142^ Further supporting this, *in vivo* and *in vitro* studies demonstrate that taurine supplementation protects retinal ganglion cells from degeneration by reducing oxidative and nitrosative stress and inhibiting apoptosis. Oral taurine increased retinal ganglion cell survival without significantly affecting intraocular pressure, suggesting a direct neuroprotective effect on retinal neurons.^143,144^ Homotaurine, a derivative of taurine, is extensively studied in glaucoma formulations. Multiple clinical trials demonstrate the efficacy of homotaurine combinations: a fixed combination of citicoline 500 mg plus homotaurine 50 mg showed beneficial effects on retinal ganglion cell function measured by pattern electroretinogram. A nutraceutical formulation (Epicolin), based on citicoline, homotaurine, epigallocatechin-3-gallate, forskolin, and vitamins, showed significant neuroprotective, antioxidant, and anti-inflammatory effects in both *in vitro* and *in vivo* studies.^145,146^

## Clinical trial developments, future directions, and conclusions

Varying levels of clinical evidence exist for a range of vitamins, minerals and nutraceuticals. The current research landscape indicates that B vitamins, particularly vitamin B_3_ (nicotinamide), represent the most advanced area of vitamin research in glaucoma, with multiple Phase II and III clinical trials ongoing worldwide. Vitamin combinations targeting metabolic dysfunction in retinal ganglion cells appear more promising than single-vitamin approaches, however few have been performed with proper single-compound-only controls which makes scientific interpretation of the studies more difficult.

The most compelling evidence exists for nicotinamide, homotaurine, resveratrol, and melatonin, whilst emerging compounds like vitamin K_1_, nicotinamide riboside, and novel compounds based on EGCG show promise for future therapeutic development (see **Box 1**). Despite this, there remains a need for more randomized controlled trials to validate and ensure the safety of supplementation for each. A key challenge for clinical trials focused on neuroprotection is the trial length and sample size required to achieve significance. Future developments in improving glaucoma clinical trial endpoints may hasten this process and further advancements in this field. A range of nutraceuticals covered here show promise to support retinal ganglion cells beyond what intraocular pressure lowering can achieve by itself to maintain functional vision for patients living with glaucoma and maintain quality of life.

Box 1. Evidence level classifications for vitamins and nutraceuticals in glaucoma

*Evidence level classifications*

*Promising-to-strong evidence*: Multiple well-designed clinical trials or meta-analyses with consistent positive results

- Homotaurine, melatonin, nicotinamide

*Promising evidence*: positive results from preliminary clinical trials or strong preclinical data with ongoing clinical development

- Coenzyme Q_10_, pyruvate, resveratrol, vitamin K_1_,

*Moderate evidence*: Some clinical data or consistent epidemiological findings, but limited scope

- alpha-lipoic acid, curcumin, forskolin, lutein/zeaxanthin, nicotinamide riboside, selenium, vitamin A, vitamin B_6_, vitamin C, vitamin D, zinc

*Emerging evidence*: Preliminary findings or metabolomic studies suggesting potential benefit

- EGCG/whole green tea polyphenols, taurine, vitamin B_9_ (folate)

*Limited evidence*: Minimal research or inclusion in formulations without specific glaucoma studies

- Copper, vitamin B_1_, vitamin B_2_, vitamin E

*Mixed/inconclusive evidence*: Conflicting results or safety concerns

- Ginkgo biloba, magnesium

*Very limited/minimal evidence*: Mentioned in literature but lacking substantial research

- Vitamin B_5_, vitamin B_7_

Given that glaucoma is a polygenic, multifactorial disease, there is unlikely to be a one-size-fits-all solution, and there is a potential that combination therapy, targeted to the right patients (*e.g.,* in those with specific genetic risk or with specific dietary deficiencies), will be more beneficial than a single supplementation therapy. For many patients, exercise and a healthy and varied diet will be sufficient to prevent the needs of excess supplementation. However, distinct forms of glaucoma are likely to benefit from different adjunctive strategies, underscoring the importance of a personalized approach that considers each patient's overall health. Accordingly, individuals interested in these interventions should consult their glaucoma specialist to determine the most appropriate course of action as most diet and supplement interventions will require rigorous, controlled clinical trials to more clearly establish their neuroprotective or functional benefits in glaucoma.
